# Sustainable Materials Enabled Terahertz Functional Devices

**DOI:** 10.1007/s40820-025-01732-1

**Published:** 2025-04-11

**Authors:** Baoning Wang, Haolan Wang, Ying Bao, Waqas Ahmad, Wenhui Geng, Yibin Ying, Wendao Xu

**Affiliations:** 1 College of Biosystems Engineering and Food Science, Zhejiang University, Hangzhou, 310058 People’s Republic of China; 2 School of Food and Biological Engineering, Jiangsu University, Zhenjiang, 212013 People’s Republic of China; 3 Zhejiang Key Laboratory of Intelligent Sensing and Robotics for Agriculture, Hangzhou, 310058 People’s Republic of China; 4 Key Laboratory of On Site Processing Equipment for Agricultural Products, Ministry of Agriculture and Rural Affairs, Hangzhou, 310058 People’s Republic of China

**Keywords:** Terahertz, Sensor, Metamaterial, Sustainable materials, Wireless communication

## Abstract

Terahertz (THz) devices, owing to their distinctive optical properties, have achieved myriad applications in diverse domains including wireless communication, medical imaging therapy, hazardous substance detection, and environmental governance. Concurrently, to mitigate the environmental impact of electronic waste generated by traditional materials, sustainable materials-based THz functional devices are being explored for further research by taking advantages of their eco-friendliness, cost-effective, enhanced safety, robust biodegradability and biocompatibility. This review focuses on the origins and distinctive biological structures of sustainable materials as well as succinctly elucidates the latest applications in THz functional device fabrication, including wireless communication devices, macromolecule detection sensors, environment monitoring sensors, and biomedical therapeutic devices. We further highlight recent applications of sustainable materials-based THz functional devices in hazardous substance detection, protein-based macromolecule detection, and environmental monitoring. Besides, this review explores the developmental prospects of integrating sustainable materials with THz functional devices, presenting their potential applications in the future.

## Highlights

- Sources and types of sustainable materials and their advantages in fabricating high performance terahertz (THz) functional devices are systematically reviewed.
- The principles and implementations of sustainable material enabled THz functional devices for wireless communication, molecular sensing, and biomedical detection.
- This review emphasizes new insights from a comprehensive analysis, presenting challenges in intelligent modulation and perception of sustainable materials assisted THz functional devices.

## Graphical abstract

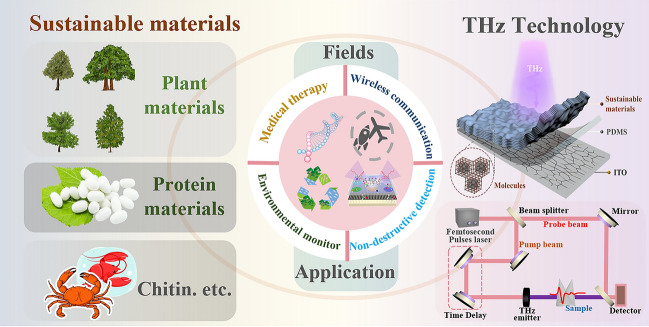

## Introduction

Terahertz (THz) waves occupy the spectrum between infrared and microwave regions. The frequency range of THz wave is 0.1–10 THz, which corresponds to 30 μm–3 mm in wavelength [1–3]. THz waves have garnered considerable applications across various fields [4], primarily attributed to their unique ability to penetrate non-conductive materials [5], inherently low photon energy levels [6], pronounced sensitivity to water and their significant capacity in communication [7, 8]. Besides, THz waves are particularly well-suited for investigating and analyzing collective vibrational and rotational modes, due to the unique spectral signatures generated by complex molecules within the THz frequency region [9, 10]. These distinctive features offer a unique perspective of the underlying molecular dynamics and contribute to the enhanced understanding of spectroscopic analysis [11]. Given the aforementioned advantages, THz waves have been incorporated into the design of functional devices such as modulators and sensors [12, 13]. Moreover, the applications based on THz functional devices have been further extended across multiple domains, including communication systems [14], biomedical technology [15, 16], food safety assessments and detection [17–19], and automated agricultural engineering [20]. This wide-scale dissemination certifies the versatile nature of THz wave technology.

Currently, the construction of THz functional devices incorporates a variety of materials including semiconductors, phase change materials, two-dimensional (2D) materials, and organic polymers, each offers distinct advantages for applicability in diverse fields. However, these materials have certain limitations, including high cost and complex process at nanoscale. For instance, conductive polymers made from organic materials have poor durability, making them susceptible to environmental factors and eventually leads to discharge issues in THz functional devices [21, 22]. To avoid excessive resource and minimize environmental impact, it is imperative to apply sustainable material in THz functional devices. Sustainable materials, with their excellent biodegradability and biocompatibility, are aptly used in the construction of THz biosensors, human health monitoring devices, and wearable sensors [23]. Additionally, their ability to actively regulate degradation time makes them suitable for medical clinical research. Furthermore, materials like cellulose, due to their inherent properties, demonstrate excellent THz wave absorption characteristics, making them effective in regulating signals within THz wave modulators and filters [24]. Thereby, those sustainable materials offer alternative choices for sustainable and environmentally friendly applications [25].

This adoption of sustainable materials not only aligns with sustainability objectives but also provides the advancement of next-generation THz functional devices [26]. Sustainable materials are typically derived from renewable resources including natural materials such as cellulose [27], chitosan [28], and silk fibroin [29], extracted from plants, marine organisms, and silkworms, respectively. Moreover, some sustainable materials, such as bio-based polymers, are derived through microbial fermentation or biosynthesis processes. Notable examples include polylactic acid, hydrocarbons, as well as various antibodies [30–32]. Those sustainable materials offer improvements in several aspects as compared to conventional chemical synthetic substances, such as environmental friendliness, biodegradability, and renewability. Besides, the abundance of natural resources offers lower production costs for the synthesis of these materials and serves as a stimulus and drive for more sustainable materials [33]. Sustainable materials have been applied in THz functional devices-based detection applications [34]. For instance, antibodies-assisted THz functional devices have achieved real-time monitoring of protein due to high sensitivity and specificity of antibodies [35]. Porous carbon aerogel materials derived from cellulose offer a high specific surface area and low loss characteristics, providing suitable solutions for fabricating THz waveguides and filters [36]. Conducting polymer aerogels-based cellulose enhanced broadband absorbed ability allowed for the intelligent design of THz devices in applications of communication devices and biomedical imaging therapy [37]. In terms of THz sensing devices, sustainable materials not only possess inherent characteristics such as hierarchical structures and morphological diversity but also provide flexible surfaces for enhanced attachment of various functional groups [38]. This capability significantly broadens the application spectrum of these functional devices across diverse fields aligning with environmentally benign THz-based sensing strategies.

**Fig. 1:**
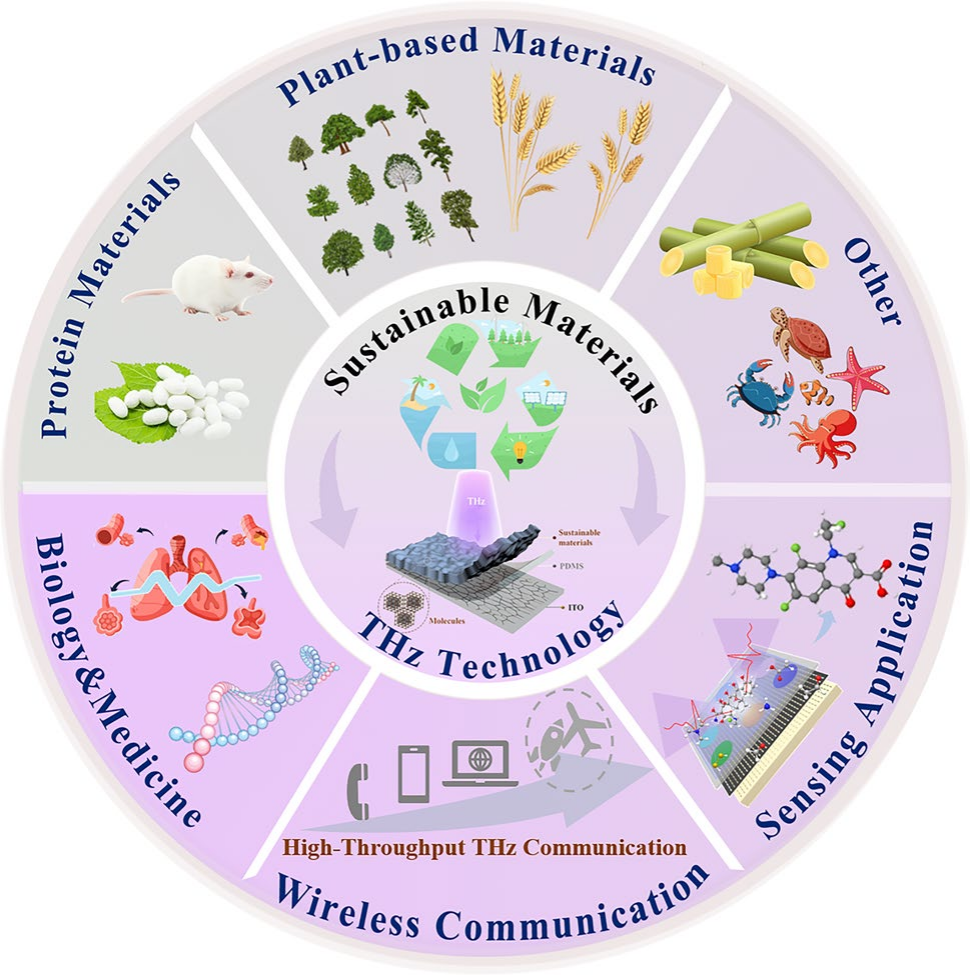
Schematic framework illustration of this review. It delivers a concept of sustainable development of THz devices based on sustainable materials. The central circular shape illustrates the sustainable materials and THz technology, while the surrounding ring indicates the source of sustainable materials and the application fields of THz functional devices

The current review encapsulates the latest advancements in THz functional devices utilizing sustainable materials in a comprehensive and highly structured manner. An overview of the scope of current work, highlighting future development trends and related concepts for THz functional devices employing sustainable materials is provided in Fig. 1. The work covers detailed origins and chemical structures of various sustainable materials, categorized into plant-based materials like cellulose, protein-based materials such as silk fibroin, and other materials like hydrocarbon polymers. The vital role of how the fundamental structures and properties of these sustainable materials help in designing and manufacturing THz devices for emerging applications is outlined. This work further discusses the roles and applications of these materials in fabricating various THz functional devices. These devices were categorized into three groups based on their research areas, including (a) transmission devices for electromagnetic interference (EMI) shields and wireless near-field communication (NFC) between different devices; (b) sensors that convert changes in humidity and micromolecules into measurable signals under varying conditions as well as environmental monitoring; (c) bio-effect devices that focus on the biological and medical applications of THz combined with sustainable materials, including the utilization of THz functional devices with natural materials in clinical biology, medical detection, and monitoring. The design and functionality of these devices are inspected along with fabrication using sustainable materials as substrates or active components. Moreover, applications of these sustainable material-based devices are covered with a primary focus on the fields of communication, microbial and macromolecular detection, environmental monitoring, biomedical, clinical treatment, etc. Finally, the challenges and prospects of devices utilizing sustainable materials are elaborated and the potential strategies for designing and constructing the next generation of THz functional devices are proposed. The current work on THz functional devices utilizing sustainable materials will play a pivotal role in constructing intelligent bio-interfaces for future applications aligned with sustainable development and environmental goals.

## Properties and Structures of Sustainable Materials

The exploration of sustainable materials is crucial for the construction of functional devices to cope with the global environmental crisis. Employing biodegradable materials will effectively reduce electronic waste (E-waste), and improve the scope and applications of sustainable materials in biomedical research and environmental monitoring. For instance, silk fibroin (SF) exhibits outstanding transparency and flexibility in THz band, which is appropriative for wearable devices [39]. Chitosan, with its antibacterial and film-forming properties, is well-suited for surface coatings of medical devices. Additionally, the excellent transmission capacity and modulation depth of cellulose enabled high performance modulator designs. Further functionalization and engineering of these sustainable materials can accelerate the development of THz functional devices, for example functionalized cellulose substrate can serve as THz imaging detectors [24]. Chemical treatment of plant-based materials can alter their THz dielectric constant and absorption characteristics, enabling dynamic control of THz functional devices. Sustainable materials have shown better performance as compared to conventional materials in the fields of communication, safety analysis, and electromagnetic sensing [40–42]. Further functionalization and engineering of these sustainable materials can lead to the development of advanced functional devices. The structures and properties of sustainable materials derived from plants, proteins, and renewable hydrocarbons are covered in this section along with distinctive features, advantages, and limitations in the fabrication of THz functional devices.

### Sustainable Materials Based on Plants

**Fig. 2:**
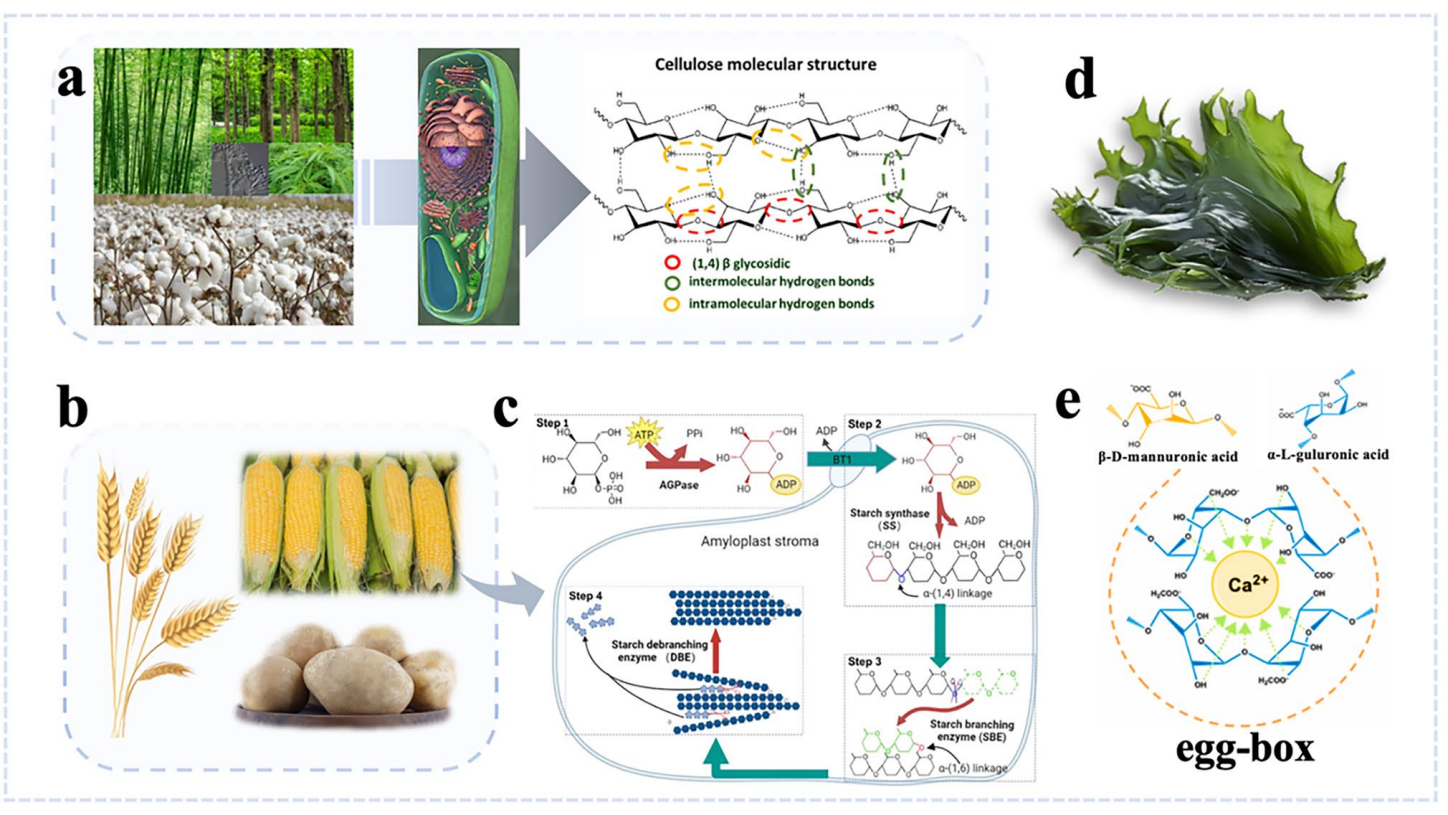
Schematic illustration of structures and morphologies of different kinds of plant-based materials. **a** Representative source of cellulose and it consists of repeating unit with the β-(1,4)-glycosidic linkage and the crystalline and disordered regions. Reproduced with permission [61]. Copyright 2023, Springer Nature. **b** Typical source of starch. **c** Chemical structure of amylose and amylopectin. Reproduced with permission [62]. Copyright 2015, Elsevier. **d** Typical source of alginate. **e** Chemical structure of β-d-mannuronic acid and α-l-guluronic acid, and graphical description of the egg-box model for alginate gelation. Reproduced with permission [58]. Copyright 2023, Elsevier

Plant-based sustainable materials, represented by cellulose, starch, chitosan, etc., have been widely studied in both structures and characteristics. Cellulose is a polymer composed of β-D-glucose pyranose units linked by β-1,4-glycosidic bonds. The linear structure of cellulose chains is stabilized by hydrogen bonds between hydroxyl and oxygen groups of adjacent ring molecules [43], as illustrated in Fig. 2a. As a ubiquitous and vital resource, cellulose is found in the cell walls of biomass sources such as bamboo [44], wood [45], cotton [46], and hemp [47]. Fungi and bacteria [48] can also synthesize renewable, cost-effective, and biodegradable cellulose. Crystalline cellulose is composed of cellulose Iα and Iβ which are in two different crystal phases. The crystal phases possess a similar pattern of hydrogen bonds, whereas intra and inter-chains crystal phases have parallel cellulose chain alignment [49]. Besides, these phases can be combined into rectangular arrays to form microfibers (MF) by parallel stacking [50]. Based on designing technologies using acid hydrolysis and mechanical treatment, cellulose can be categorized as cellulose nanofibers (CNFs), cellulose nanocrystals (CNCs), and bacterial cellulose (BC). The structure of CNCs closely resembles that of CNFs, differing primarily in morphology and crystallinity whereas CNCs exclusively exhibit crystalline properties [51]. Compared to CNFs and CNCs, BC has a distinct nanostructure with better mechanical strength. Innovations in nanotechnology have enabled the creation of cellulose and its derivatives through physical or chemical modification methods, resulting in products like films, aerogels, nanofibers, and carbon fiber-based nanocellulose. Currently, cellulose has been applied in sensing research due to its inherent electroactive behavior and dielectric properties [52]. Starch, derived from corn, potato, cassava, and other crops is a natural plant-based material, as shown in Fig. 2b [53]. The structure of starch primarily consists of glucose units linked by glycosidic bonds and includes two main molecular forms: amylose (linear starch) and amylopectin (branched starch). Amylose is connected by α-1,4-glycosidic bonds, whereas the main chain of amylopectin is linked by α-1,6-glycosidic bonds (Fig. 2c). These structural variations lead to differences in physical properties and functional traits, such as water solubility, modification ease, and susceptibility to enzymatic hydrolysis [54]. The practical application of starch, particularly in constructing THz functional devices, requires enhanced properties [55]. Starch polymers form strong hydrogen bonds due to hydroxyl groups on the chain, complicating processing and resulting in suboptimal physical properties of starch film sensing. To address these issues, designed modification methods such as cross-linking and molecular substitution have been explored [56]. Moreover, starch is non-toxic, transparent, flexible, and biodegradable, making it an excellent sustainable material for THz functional devices. Alginate is another sustainable material derived from marine brown algae, which constitute the primary components of their cytoplasm and intercellular matrix [57]. It holds biodegradability like other sustainable materials and possesses the abilities of non-toxic, highly adhesive, and flexible. Besides, the flexibility of alginate is primarily governed by the content of α-l-guluronic acid (G) and β-d-mannuronic acid (M) residues (Fig. 2d) [58]. These unique properties make alginate highly suitable for manufacturing thickeners, emulsifiers, and films. In gel-based materials, alginate forms an egg-box structure with certain cations through cross-linking reactions [59]. Cations like calcium ions are trapped in cavities of the gel network, forming a spiral conformation (Fig. 2e). In addition, the hardness and elasticity of gel can be modified by adjusting the proportion of M-G blocks, which can be applied in biosensing and medical applications according to the designed requirements [60].

### Sustainable Materials Based on Proteins and Peptides

Proteins exhibit diverse structures, each accorded with specific biological functions. Protein-based sustainable materials have shown promise due to their excellent biodegradability, renewability, and wide range of applications. The protein-based sustainable materials are covered with key categories such as silk proteins, antibodies, and collagen. Silk protein, celebrated for its outstanding mechanical properties and biodegradability, is increasingly utilized in biomedical and sensing applications. Additionally, the precise engineering of antibodies is crucial for developing diagnostic tools and therapeutic agents. Collagen, known for its superior biocompatibility and strength, is widely used in medical and cosmetic fields. Thus, the protein-derived materials are elucidated for their potential in advancing sustainable technological progress.

#### Silk Fibroin

**Fig. 3:**
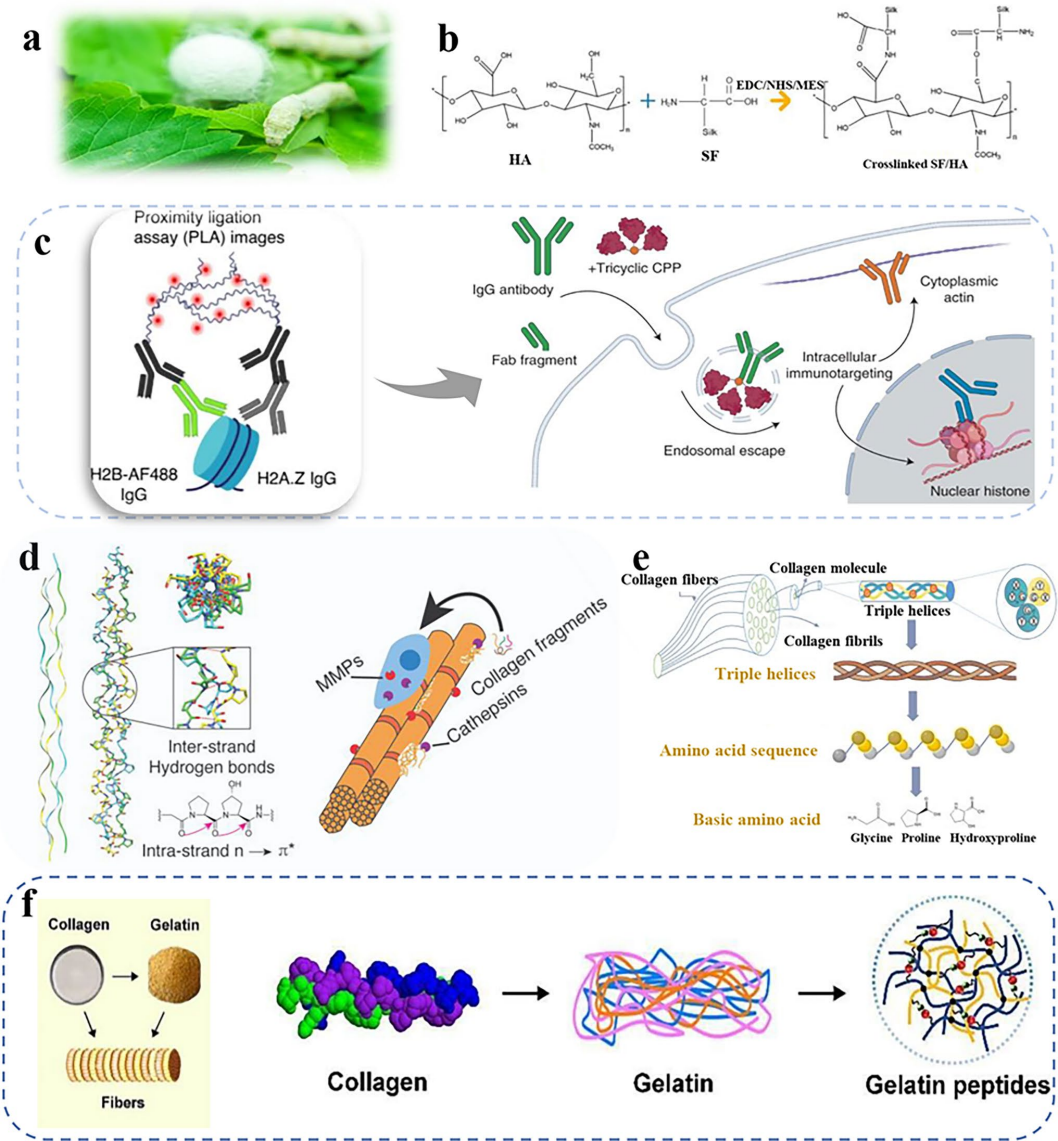
Schematic illustration of structures and morphologies of different kinds of protein-based materials. **a** Structure of bombyx mori silk cocoons and fibroin. **b** Multifunctional silk proteins and cross-linking reactions. Reproduced with permission [86]. Copyright 2024, Elsevier. **c** Y-shaped architecture of antibodies and highly specific stem region or Fc fragment. Reproduced with permission [70]. Copyright 2023, Springer Nature. **d** Triple helix structure of collagen with intrastrand n → π* interactions by the trans-amide bond conformation and interstrand hydrogen bonds by glycine residues in the helical core. Reproduced with permission [87]. Copyright 2024, American Chemical Society. **e** Collagen fibrils comprising three basic amino acids proline, glycine, and hydroxyproline. Reproduced with permission [88]. Copyright 2024, Elsevier. **f** Structure of collagen and gelatin. Reproduced with permission [89]. Copyright 2024, Elsevier

Silk protein is a type of natural fiber primarily secreted by spiders, silkworms, and other organisms. Silkworm primarily produces SF and sericin fibroin in the silk gland (Fig. 3a) [63]. SF has garnered significant research attention and found widespread applications in medical practice and biological tissue engineering [64]. It exhibits two distinct structures, namely silk I (β-folded conformation), and silk II (non-β-folded structure). Both consist mainly of heavy chains (H, 390 kDa) and light chains (L, 26 kDa) connected by disulfide bonds [65]. The tyrosine and serine-rich sequences in silk I enhance molecular cross-linking, and create regions to improve strength and toughness. Furthermore, these amino acid sequences provide a versatile platform for the attachment and modification of functional groups including –OH, COOH, and –NH_2_ groups (Fig. 3b) [66]. The silk II structure, constituting the SF region, contributes to the softness and breathability of SF. Notably, SF’s biocompatibility renders it an ideal candidate for applications in tissue engineering and drug delivery systems. Its outstanding mechanical strength and biodegradability make it a promising choice for sustainable composite materials and functional device applications [67].

#### Antibodies and Aptamers

Antibodies and aptamers, as biological recognition elements, exhibit exceptionally high specificity and binding affinity toward their target molecules to enable precise binding interactions. Antibodies originate from B-cell differentiation, whereas aptamers are synthesized and selected via advanced in vitro systematic evolution of ligands by exponential enrichment (SELEX) method, a technique that systematically selects ligands through exponential enrichment [68, 69]. Antibodies adopt a Y-shaped architecture, with two heavy chains and two light chains interconnected by disulfide bridges to form their distinct structure. The ends of the Y-shape contain antigen-binding sites (Fab fragments) responsible for antigen specificity, while the stem (Fc fragment) handles immune system functions, as shown in Fig. 3c [70]. On the other hand, aptamers consist of single-stranded oligonucleotides (e.g., ssDNA or RNA) that fold into intricate three-dimensional (3D) structures, tailored for specific molecular recognition [71]. Both antibodies and aptamers exhibit remarkable specificity and provide the precision required in biological detection methodologies with robust stability, maintaining their structural integrity and functionality across a broad spectrum of varying temperatures and pH levels [72]. These biomolecules can be chemically modified to attain multifunctionality and versatility [73]. These attributes enable sustainable antibodies and aptamers with vast potential for widespread adoption in biomedical fields including disease diagnostics, drug discovery, environmental monitoring, and the detection of hazardous substances.

#### Collagen

Collagen is the most abundant protein prevalent in connective tissues (skin, bones, cartilage, tendons, ligaments) [74], which is secreted by fibroblasts and other cells. Collagen molecules exhibit a 3D spiral structure, comprising three α polypeptide chains that interact via hydrogen and covalent bonding (Fig. 3d) [75]. The stability of this 3D spiral structure is attributed to the composition of each α chain, primarily consisting of glycine, proline, and hydroxyproline. Furthermore, mechanical stress displaces hydrogen bonds, causing dipole moments to align along collagen molecules, which leads to the occurrence of permanent polarization (Fig. 3e) [76]. These collagen molecules with 3D spiral structures self-assemble into fibrils, which subsequently aggregate to form collagen fibers [77]. The unique composition of these structures imparts mechanical strength and elasticity, crucial for tissue repair, structural support, and cell adhesion across vertebrates and invertebrates [78]. As a sustainable material, collagen exhibits excellent biocompatibility and degradability and can be tailored for desired purposes through chemical or physical technologies. Its 3D spiral structure and self-assembling fibers make it an ideal sustainable material for clinical applications, particularly in cartilage repair and bone transplantation [79]. Collagens can be hydrolyzed into gelatin, an irregular blend of shortened and disordered polypeptide chains. Despite its similar composition gelatin is characterized by shorter and randomly arranged polypeptide chains (Fig. 3f) [80]. Gelatin is primarily sourced from animal skins and bones, particularly pigs and cows, via acid or alkaline processing, whereas a minor fraction is derived from fish [81]. Gelatin-based films have certain limitations, such as their ability to absorb moisture in humid conditions. However, drug-loaded microcapsules and nanoparticles allow precise release control and targeted delivery, to enable sustainable platforms for cell culture substrates and scaffolds in regenerative medicine and tissue engineering [82]. Collagen and gelatin, as vital proteinaceous materials, possess ubiquitous applications in food and biomedical sectors due to their biocompatibility [83], biodegradability [84], and favorable mechanical characteristics [85].

### Others

**Fig. 4:**
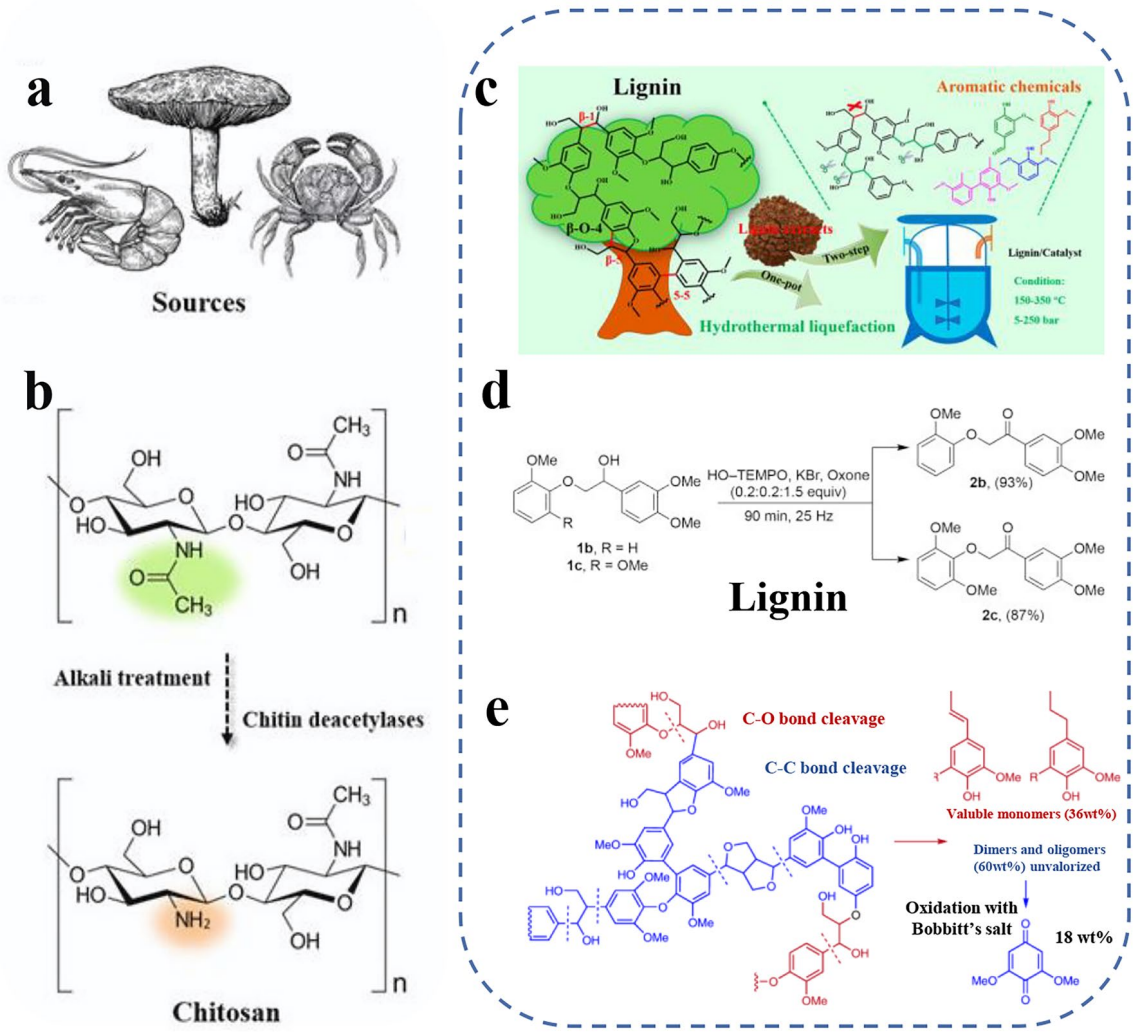
Schematic illustration of other sustainable materials. **a** Various chitosan sources. **b** Conversion from chitin to chitosan by chemical or enzymatic deacetylation process. Reproduced with permission [102]. Copyright 2024, Frontiers Media S.A. **c** Resource and structure of lignin, the construction of aromatic chemicals. Reproduced with permission [103]. Copyright 2020, American Chemical Society. **d** Aromatic monomers of intricate 3D network architecture. Reproduced with permission [98]. Copyright 2018, American Chemical Society. **e** Lignin’s diverse functional groups and various derived functional materials. Reproduced with permission [97]. Copyright 2021, Springer Nature

Chitin, a widely abundant natural polysaccharide on earth, is an excellent biomass sustainable material for constructing functional THz devices. It is primarily composed of repeating units of N-acetylglucosamine linked by β-1,4-glycosidic bonds [90]. Due to interactions between hydrogen bonds and adjacent functional groups including –NH and –OH, chitin exhibits hydrophobic properties and can be completely dissolved by chitinase, leaving no harmful residues and posing no environmental threat [91]. Generally, chitin is extracted from shell waste of crustaceans like crab and shrimp shells, as well as exoskeleton materials of invertebrates such as squid bones (Fig. 4a); it can also be sourced from fungi and bacteria [90, 92]. It has been successfully utilized to generate films for sensing applications and designed as hydrogels with excellent conductivity for biomedical and mechanical applications due to its unique biochemical properties [93]. Chitosan deacetylases are commonly employed to enzymatically deacetylate chitin by chemical or physical modification to expand its applications. As demonstrated in Fig. 4b, this process substitutes acetyl groups with amino groups to enhance the hydrophilicity and enable a better understanding of the biological activity mechanisms of its degradation products [94]. Additionally, aminated chitosan more readily combines with functionalized materials, broadening its application scope, particularly in biomedicine. The achieved chitosan polymer exhibits antibacterial and antioxidant properties, and its self-assembly capability holds significant potential in sensing and clinical treatment applications [95]. Lignin, a vital component of plant cell walls, is a complex polymeric material abundantly prevalent in wood, herbaceous, and other higher plant species (Fig. 4c) [96]. Lignin is predominantly sourced from chemical extraction or biodegradation of wood and plant byproducts, exhibiting significant heterogeneity and structural irregularity [48]. It consists of aromatic monomers, such as p-coumarin, guaiacol, and syringol, and randomly polymerizes via β-O-4 and C–C linkages to form a 3D network architecture, as depicted in Fig. 4d [97, 98]. This intricate structure confers lignin with remarkable mechanical properties. Furthermore, lignin's ability to interface with cellulose and hemicellulose results in composite materials that exhibit hydrophobic properties, minimizing water loss and facilitating efficient water and nutrient transport [99]. Additionally, lignin’s sustainability makes it a viable option for biomass energy. It can be readily converted to a diverse array of chemicals including phenolic, aromatic compounds, and polymer monomers, which hold promising industrial applications [100, 101]. Moreover, its diverse functional groups provide a versatile platform for targeted chemical modifications and the formation of a range of functional materials, as shown in Fig. 4e.

### Advantages and Disadvantages of Sustainable Materials

Sustainable materials, such as plant-derived materials, protein-based composites, and chitosan derivatives, demonstrate structural versatility and superior biological attributes. These features include abundant availability, renewability, biodegradability, low environmental footprint, and multifunctionality [32]. Sustainable materials-based THz functional devices hold exceptional promise for fabricating flexible, transparent, and biocompatible components, leveraging their inherent compatibility with biological systems and electromagnetic wave modulation capabilities [52]. This cooperation enables novel device architectures that significantly reduce reliance on conventional synthetic materials while simultaneously fulfilling the stringent requirements for biomedical sensing and adaptive photonic systems. Despite these promising attributes, their robust applications in THz functional devices still faces challenges, such as poor conductivity, inadequate stability, and complex processing, as shown in Table 1. To overcome these limitations, materials such as plant-based, protein-based, and chitosan derivatives are combined with high-performance materials (such as graphene, carbon nanotubes, metal oxides) to form high-quality sustainable hybrid composite materials [78]. Thus, the mechanical properties, conductivity, and THz wave transmission efficiencies are improved. Additionally, by designing porous metamaterial structures, the THz response characteristics of materials can be further optimized.

**Table 1:** Advantages and disadvantages of devices based on different sustainable materials

Sustainable materials	Advantages	Disadvantages
Cellulose	Rich sources, including CNFs, CNCs, BC; Low dielectric loss	Strong moisture absorption; Easy to contaminate impurities
Starch	Easy to process into thin films or 3D structures on surface of THz devices	Poor mechanical performance; High dielectric loss
Alginate	Easy to form flexible hydrogels for THz device packaging and biomedical applications	Poor conductivity; High absorption loss
Silk fibroin	Excellent mechanical properties and optical transparency, suitable for THz transmission	Complex extraction and purification processes; High humidity sensitivity
Antibodies	High sensitivity and selectivity; Surface functionalization to enhance local field effects	Poor stability; Expensive production and purification
Collagen	Excellent flexibility and stretchability; Suitable for tunable THz devices	High humidity sensitivity; Dielectric coefficients are easily affected
Chitin/Chitosan	Natural antibacterial properties; Outstanding film-forming properties	High dielectric loss
Lignin	Excellent UV shielding performance to protect the photosensitive components of THz devices	Complex aromatic structures lead to high dielectric losses

In addition, the use of sustainable materials as substrates or packaging components for THz functional devices can lead to high solubility in water/biological fluids due to their excellent biodegradability. Especially, it can lead to a reduction in the mechanical strength of THz functional devices, which is hard to meet the demands for high strength and durability. As a result, those sustainable materials present high dielectric loss as well as poor performance in THz wave propagation, modulation, and sensing. A plausible solution is to fabricate an encapsulation layer, which is crucial for prolonging the service life of the functional devices. Currently, copolymers like poly(lactic-*co*-glycolic acid) [104] and SF are commonly employed in encapsulation layer preparation, offering environmental stability and interface adaptability. These strategies provide potential multi-scenario applications based on THz functional devices.

## THz Communication

THz waves provide higher communication frequency and bandwidth comparing with radio waves [105], enabling speedy data transmission and suitable for transmission requirements of high throughput data. In addition, the penetrability and directionality of THz waves can protect user privacy [106]. Compared to traditional materials-based THz functional devices, the utilization of sustainable materials can reduce E-waste due to its degradation ability in the environment and ensure that the equipment can maintain stability during high-frequency operation due to its excellent thermal conductivity.

### Electromagnetic Interference Shielding and Absorption

**Fig. 5:**
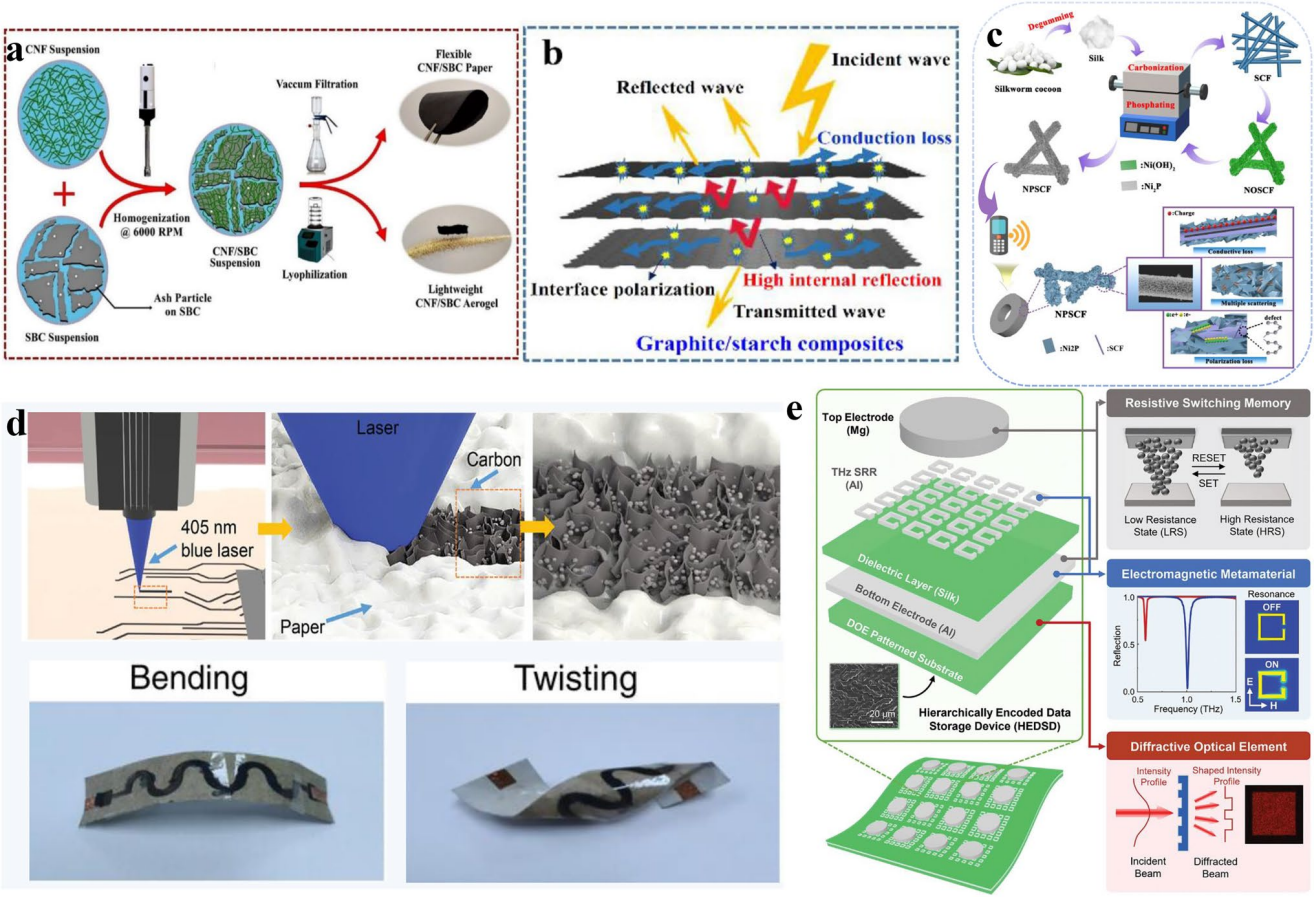
Schematic illustration of the EMI and wireless communication field based on sustainable materials. **a** Fabrication process of paper and aerogel-based CNFs-sustainable biocarbon as well as those of identification mechanism. Reproduced with permission [34]. Copyright 2023, Elsevier. **b** Preparation of graphite/starch composites and EMI shielding mechanism. Reproduced with permission [111]. Copyright 2022, Chinese Society of Metals. **c** Aerogel-based on 2D Ni_2_P nano sheet anchored on 1D silk-derived carbon fiber. Reproduced with permission [112]. Copyright 2022, Chinese Society of Metals. **d** Stacked 3D carbon/cellulose composite layer as THz shielding material in wearable energy storage devices. Reproduced with permission [136]. Copyright 2022, Wiley. **e** Design and application of implantable data storage systems. Reproduced with permission [134]. Copyright 2022, Wiley

The electromagnetic interference shielding efficiency (EMI SE) of designed materials is primarily attributed to their abundant surface functional groups and high conductivity [107]. This is mainly due to the weak interaction between nanosheets induced surface plasmon resonance, promoting the absorption of electromagnetic waves, and therefore demonstrates that the EMI SE is related to the film thickness of the material [108, 109]. To ensure the stability of signal transmission, alternative materials with excellent EMI SE is crucial. Sustainable materials have certain prospects in the field of THz communication due to their excellent electromagnetic properties, mechanical strength, and toughness (Table 2). Cellulose, widely used in EMI shielding, provides sufficient structural support for functional devices. Krisztian’s group fabricated a CNF film-based microstructure device, which is highly porous, lightweight, as well as extremely low relative permittivity, providing radio frequency filters operating at sub-THz regime [110]. Numerous studies on cellulose for THz EMI shielding still necessitate the use of traditional polymers like poly (methyl methacrylate) (PMMA) and polyvinylidene fluoride (PVDF), which can pose environmental risks. To decrease the risks, Avinash et al. used CNFs with highly conductive sustainable biocarbon (SBC) derived entirely from biomass through vacuum filtration and freeze-drying technique to generate a 3D porous ultralight aerogel with a thickness of 3 mm and flexible nanopaper with a thickness of 0.6 mm (Fig. 5a). The graphite SBC structure provides high conductivity, while the CNFs impart mechanical robustness. The results demonstrate that the aerogel and ultrathin paper devices exhibit excellent electromagnetic interference shielding effectiveness, achieving 70 and 46 dB, respectively [34]. A graphite/starch slurry formed by ultrasound treatment improves the delamination and dispersion performance of graphite in starch through cation-π interaction. The enhancement of EMI shielding performance from 17.4 to 66.8 dB was assigned to the Salisbury screen effect (Fig. 5b) [111]. Besides plant-based materials, silk is extensively utilized in EMI shielding for THz communication. Pan et al. [112] anchored 2D Ni_2_P nanosheets on 1D silk-derived carbon fiber to form a resistor-dielectric type absorber. A controllable pyrolyzation strategy and the disproportionated reaction were employed to achieve a maximum reflection loss value of − 56.9 dB and demonstrate enhanced EMI shielding. Additionally, the impact of aerogel thickness on electromagnetic absorption was examined, as depicted in Fig. 5c. In order to improve the shielding performance of THz functional devices derived from sustainable materials, it is necessary to combine different mechanisms or study composite devices combined with different natural sustainable materials [113]. Haataja team has designed a composite sensor by preparing a mixed frozen gel that combines cellulose nanofibers, polyvinyl alcohol (PVA), and Ti_3_C_2_TX MXene. Employing a temperature gradient freeze-drying method, a honeycomb and layered pore structure is produced. PVA enhances the gel's structure, and optimized MXene content helps in improving the durability and compression strength. This composite gel device demonstrates the role of sustainable materials in THz wave absorption through synergistic effects of dielectric constant and EMI shielding, which is improved by 46 dB after a sample attenuation of 50 wt%, significantly surpassing commercial EMI shielding standards [114]. Similarly, Tao et al. developed a THz composite device using cellulose and its derivatives to switch an active amorphous carbon nanomaterial. As demonstrated in Fig. 5d, the stacked 3D carbon/cellulose composite layer integrates deformable mechanical properties, ultra-high conductivity, and THz shielding capabilities to achieve more precise sensing results. Sustainable materials can play an important role in THz EMI shielding due to their unique characteristics. Other sustainable materials such as chitosan, gelatin and lignin [115, 116] also have certain advantages in this field and have been recorded in Table 2.

**Table 2:** Summary of THz technologies based on sustainable materials in the application of EMI shielding

Sustainable materials	Functional materials	Transduce mechanism	Results	References
Nanofibrillar cellulose	Aerogels	Carbon nanotube and cellulose aerogels	66.0 dB	[117]
CNFs film	CNFs film and silica foams	Sol–gel method	–	[110]
CNFs	CNFs and PVDF-PMMA	Air spray coating	20.0 dB	[118]
BC	BC-rGO aerogel	Freeze-drying and pyrolysis	− 46.1 dB	[119]
Cellulose	Cellulose composite aerogel	–	20.8 dB	[120]
Chitosan	Carbon porous aerogel	Dipole polarization and interface polarization	− 52 dB	[121]
NFs	CNFs and nylon	Air spray coating	44.0 dB	[122]
CNFs&SBC	Aerogels and nanopapers	Vacuum filtration and freeze-drying	46.0 and 70.0 dB	[34]
Starch	Graphite and starch slurry	Cation–π interaction	66.8 dB	[111]
Alginate	GCoAMs	Spraying and crosslinking coagulation	− 70.4 dB	[123]
SA	SA and CNTs PCMs	A controlled directional freezing method	− 48.0 dB	[124]
SCF	SCF-2D Ni_2_P	Grown vertically and cross-linked	− 56.9 dB	[112]
Silk fiber	SiC fiber aerogel	Carbothermal reduction	68.0 dB	[125]
Collagen	metal–organic hydrogels and porous aerogel	Magnetic coupling effect	− 85.0 dB	[126]
Gelatin	Gelatin and CNT-SiC film	Chemical vapor deposition	–	[127]
Alginate	Alginate derived carbon composites	Freeze-drying and carbonization	− 40.2 dB/5.4 GHz	[128]

*PVDF* polyvinylidene fluoride, *PMMA* poly (methyl methacrylate), *SBC* sustainable biocarbon, *BC-rGO* Bacterial cellulose-derived C/thermal reduced GO, *GCoAMs* cobalt@graphene aerogel microspheres, *SA* sodium alginate, *CNTs* carbon nanotubes, *PCMs* porous composite materials, *SCF* silk-derived carbon fiber

### Wireless Communication

The advances in wireless transmission technology hold significant potential for THz technology in upcoming 6th generation mobile networks (6G) communication systems [129, 130]. Since 6G will feature an extremely large-scale antenna array (ELAA), the substantial increase in both antenna numbers and carrier frequencies will expand the near-field region of ELAA by several orders of magnitude to warrant enhanced multi-user communication [131]. Besides, NFC is a short-range wireless technology that enables data exchange between electronic devices within a few centimeters. NFC is primarily utilized in applications such as payment systems, identity verification, and data transfer [132]. Given that the number of communication paths typically falls short of the antenna’s number, it is crucial to identify a channel path with a low pilot, design an appropriate codebook, and convert the channel into a sparse representation. Traditional materials used in THz communication devices can pose environmental risks and complicate heat dissipation management during the process of channel conversion, particularly for NFC modules in wearable and medical implant devices [133]. Conversely, the biocompatibility and safety of sustainable materials offer a promising foundation in THz communication.

THz technology has been applied in the construction of communication devices due to the design of a switchable broadband THz absorber. Kuang et al. [37] designed a conducting polymer-cellulose aerogel and attained a THz transmission of 13%–91% with a specular reflection loss of less than − 30 dB. Besides, surface hydrophilicity, de-icing, and de-frosting properties were evaluated to provide a pathway for designing tunable attenuators or shields for wireless electronic devices. Similarly, Wei et al. [134] proposed a hierarchically encoded data storage device (HEDSD) based on silk for photonic information capture by integrating THz metamaterial device, derived from encoded photons or electromagnetic information by inducing different resonant states. Due to excellent electrical properties and controlled transiency, silk-based HEDSD could simultaneously collect electronic and optical information by cooperating with a resistive switching memory and a diffractive optical element, respectively. The in vivo degradation studies in mice have demonstrated the effective implantation and biodegradation of silk-based HEDSD, to be applicable for implantable data storage systems, as depicted in Fig. 5e. Electronic and photonic devices comprising operational circuits and programmable THz encoded by silk-based metamaterials have been reported that can undergo controlled physical degradation in specific environments and enable time control of light distribution irradiated on the device with optimized diffraction optical elements and hence achieve precise light distribution [135]. As analyzed from multiple perspectives, sustainable materials such as cellulose, SF, and chitosan demonstrate significant potential and advantages in the realm of NFC combined with THz technology. This synergy not only enhances device performance but also aligns with environmental and sustainable development objectives to advance NFC technology.

## THz Sensing

THz sensing devices fabricated using sustainable bio-origin materials exhibit extensive applications across various fields due to their biocompatibility and environmental friendliness. Sustainable natural materials, such as plant-based materials and protein materials, could bring new possibilities in humidity sensing, safety detection, and environmental monitoring. This is primarily attributed to the fact that molecular vibrational frequencies of certain materials are located in the THz band, presenting fingerprint absorption in this regime [137, 138]. This enables the detection of molecular structures and physical properties of materials and thus facilitate THz sensing. Furthermore, the THz transmission and scattering behavior of materials are highly dependent on their microstructural characteristics [139]. For instance, porous materials and nanostructures derived from cellulose significantly affect the propagation pathways and velocity of THz waves [140]. Therefore, functional devices incorporating sustainable materials enable real-time, accurate, and non-destructive THz sensing, owing to THz waves’ excellent penetration, safe, benign nature, and high resolution.

### Humidity Sensing

**Fig. 6:**
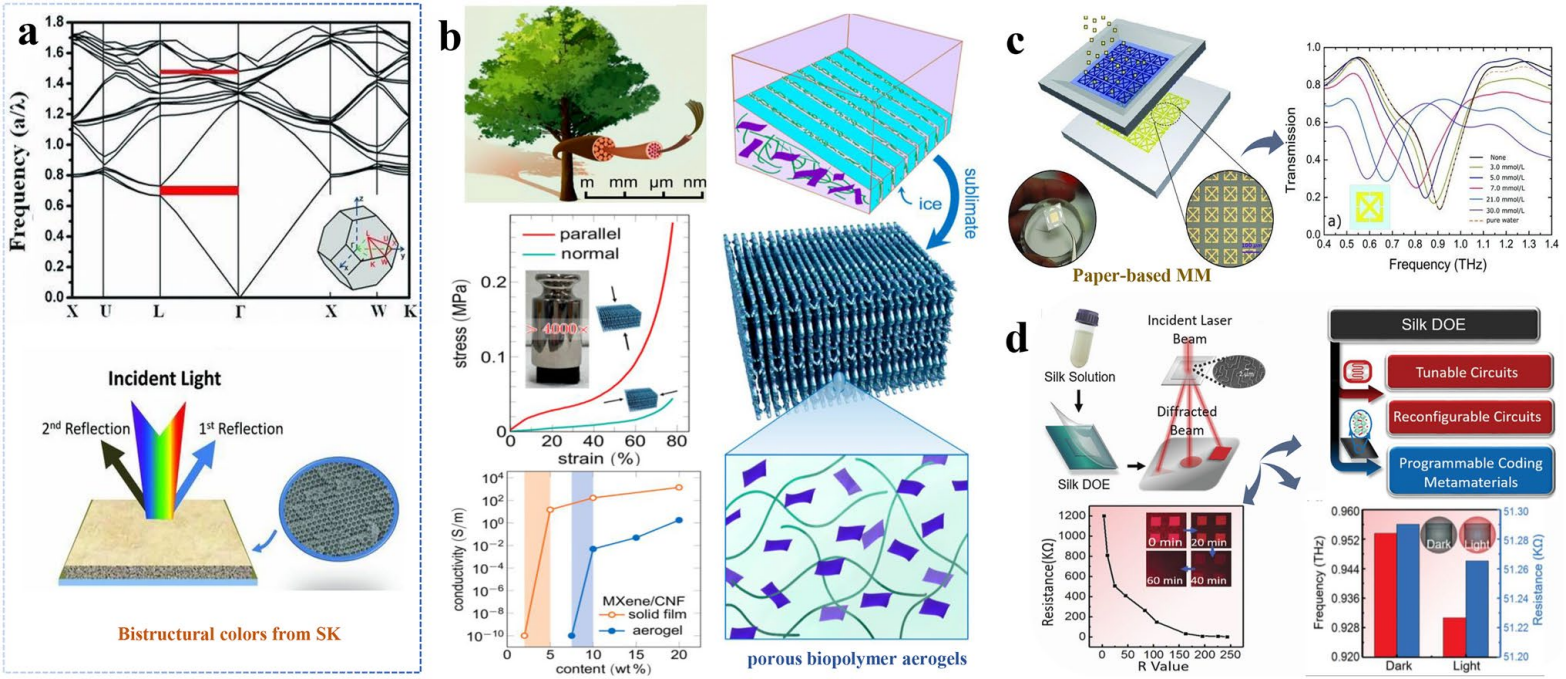
Sensing based on various sustainable materials. **a** Photonic band structure of a silk fibroin inverse opal along the high-symmetrical directions. Reproduced with permission [145]. Copyright 2013, Wiley. **b** Construction of lightweight cellulose nanofiber-based lamellar porous biopolymer aerogels and identification of organic gas under a low density [158]. Copyright 2021, American Chemical Society. **c** THz sensing platform based on paper with metamaterials for monitoring different concentrations of glucose. Reproduced with permission [157]. Copyright 2011, Wiley. **d** A type of electronic and photonic devices composed of programmable THz encoded by silk-based metamaterials. Reproduced with permission [135]. Copyright 2020, Wiley

Humidity sensing technologies have received considerable interest due to their precise monitoring, rapid response, and environmental stability [141–143]. As opposed to conventional porous ceramics, sustainable materials display pronounced hygroscopic properties. Changes in material conductivity or capacitance in response to environmental humidity are enhanced by THz technology, leading to significant improvements in the sensitivity and accuracy of humidity sensors. Jin et al. [144] designed a THz humidity sensor by coating silk fibroin solution on the surface of metamaterial fabricated by laser micro-drilling technology. The fabricated THz humidity sensor showed high humidity sensitivity by exploiting the high affinity of substrate for water molecules. The sensor achieved 0.20 GHz more in frequency shift than sensing on a metamaterial only. Besides, SF-based materials can be conjugated with other materials for the fabrication of humidity sensors. For instance, Diao et al. [145] proposed bistructural colors based on inverse opal structural SF photonic crystals. The results indicated that two reflection peaks and their separation can be attenuated by adjusting the lattice constant of designed structure. Additionally, the reflection peaks of silk fibroin inverse opal can be altered with the changes in humidity levels (Fig. 6a). Moreover, the humidity sensor with SF and THz metamaterials shows a linear relationship between humidity and additional resonance shifts [146]. In order to further develop intensity-interrogated THz humidity sensors, You and coworkers [147] constructed a hydrophilic surface layer coated by polymer. This fabricated humidity sensor could effectively absorb water vapor after surface modification using UV-induced grafting and polymerization, which achieved a low limit of detection (LOD) of 1.0 GHz ranging from 25 to 99% relative humidity (RH). The integration of THz technology and sustainable materials, such as protein-based materials and polymers provide potential promise for humidity sensing. These sustainable materials, known for their excellent hygroscopic properties, can sense environmental humidity through changes in their conductivity or capacitance [148]. Moreover, the integration of THz surface plasmonics enables high sensitivity and accuracy of humidity sensors for sub-trace level changes, as summarized in Table 3. These humidity sensors have a broader application prospect in practical applications, such as environmental monitoring, medical health, and food safety.

**Table 3:** Summary of THz sensing applications based on sustainable materials

Sensing types	Sensing materials	Mechanism/methods	Target	Linear range	LOD	References
Humidity sensing	SF	Micro-drilling	H_2_O	4–76.1% RH	0.11 GHz	[144]
	SF	Photonic	H_2_O	30–80% RH	0.20 GHz	[145]
	Polymer	Diode module	H_2_O	25–99% RH	1.00 GHz	[147]
	Gelatin	Gelatin-coated chalcogenide-silica	H_2_O	–	1.30 THz	[149]
	Cellulose	TOCFs/CNTs	–	11%–95%	–	[150]
	CNFs	CPW	–	55%–100%	2.82 MHz	[151]
Environmental hazard factors monitoring	Chitosan	Hybrid hydrogels	AB113	–	7.80 µg	[152]
	SA	BC-G-SA	–	Bacteriostat 50–200 g mL^−1^	–	[153]
	Epoxy	Graphene/epoxy composite aerogels	Electromagnetic pollution	–	–	[154]
Macromolecules detection	Cellulose	Organometallic perovskite and CE mixed film	–	0–30% CE	–	[155]
	SF	SF with graphene	Protein	–	0.35 ng mL^−1^	[156]
	Cellulose	Paper-based MM device	Glucose	–	14.30 GHz/mmol L^−1^	[157]

*TOCFs/CNTs* 2,2,6,6-tetramethylpiperidine-1-oxyl oxidized cellulose fibers/carbon nanotubes, *CPW* coplanar waveguide technology, *GNP* gold nanoparticles, *EGFR* epidermal growth factor receptor, *CE* cellulose ester, *BC-G-SA* biochar cross-linked glutaraldehyde with sodium alginate, *AB113* acid blue 113 azo dye, *MM* metamaterials

### Environmental Hazard Factors Monitoring

Environmental monitoring utilized by THz functional devices with sustainable materials due to these substantial advantages as listed: (1) THz waves possess unique spectral characteristics and strong penetrative capabilities allowing for internal component analysis without sample damage to enable reliable and suitable monitoring of soil, water, and air samples [159]. (2) THz technology provides fast and real-time environmental monitoring and acquires timely information on pollutant concentration and distribution, indicating a broad application prospect [160]. To prevent environmental waste generated by THz sensing devices, sustainable environmentally-friendly materials are strongly required. In environmental safety detection, accurate monitoring of certain harmful pollutant gases and anthropogenic factors is critical for environmental safety management. Additionally, high-performance, low-cost, and multi-functional THz sensors are of great importance in environment monitoring and governance. Gustav’s team [158] developed a method for the construction of lightweight cellulose nanofiber-based lamellar porous biopolymer aerogels. As presented in Fig. 6b, by embedding MXene layers including AgNWs and CNF into the aerogel matrix, the composite aerogels achieve efficient THz birefringence with a low density of 2.7 mg cm^−3^. This demonstrates its application in organic gas recognition and monitoring. The THz functional devices, utilizing a biopolymer aerogel with 3D porous structures, exhibited significant potential in identifying harmful gases and monitoring the environment. Liu and coworkers [154] prepared a type of anisotropic graphene/epoxy composite aerogels, which provided a way to decrease the electromagnetic pollution problem. Moreover, aerogels-based composite prepared by other technologies can also solve electromagnetic pollution. For example, Wan et al. [161] fabricated an ultralight cellulose fiber/thermally reduced graphene oxide hybrid aerogel using lyophilization and carbonization technology, which possessed excellent mechanical resilience and cycling stability. Thus, it can be concluded that sustainable materials-enabled THz sensors are promising for harmonic practical applications [162].

### Macromolecules Detection

In the field of safety detection, the combination of THz technology and novel sustainable materials enables non-destructive detection of related substances. For instance, the identification and quantification of harmful substances can be achieved by detecting the attenuation and phase changes of THz waves when passing through sustainable materials [37]. The unique properties of THz technology establish it as an innovative detection method. Its high sensitivity to weak molecular signals broadens its applications in biomolecule studies. Additionally, the distinct position of THz spectroscopy lays a foundation for advancing sensing concepts across other frequency bands [1, 163]. Although THz sensors fabricated from conventional materials can achieve ultra-low detection limits for harmful substances, the non-degradability of these materials poses environmental concerns [164]. Currently, sustainable materials are being studied due to their biodegradability and diversity. Integrating THz technology with sustainable materials provides the detection of macromolecules such as proteins, synthetic materials, and smaller entities such as bacteria, viruses, heavy metals, pesticides, and antibiotics [17, 165–167]. Multifunctional properties of sustainable materials for enhanced hazardous gas detection have also been reported [138]. For the detection of macromolecular substances, THz technology offers exceptional sensitivity and resolution in detecting macromolecular structures. Its spectral signatures reflect the distinct vibrational and rotational energy levels of large molecules (proteins), and provide a unique molecular fingerprint for precise identification and quantitative analysis [168]. Tao et al. [157] designed a THz sensing platform based on a paper with metamaterials, which can serve as a detector for monitoring different concentrations of glucose through resonance shift (Fig. 6c). This paper-based metamaterials can achieve a sensitivity of 14.3 GHz/mmol L^−1^ for glucose. In addition, Huang et al. [156] proposed a THz meta-surface biosensor for the highly sensitive detection of trace proteins incorporated with optimal silk protein concentrations in graphene to result in 0.35 ng mL^−1^ sensitivity through analyzing Fermi level. As depicted in Fig. 6d, the silk protein content was optimized to improve conditions for THz sensing devices. However, the sensitivity and specificity of THz technology for molecular detection require further enhancement. Recent advancements in the utilization of aptamers and antibodies have demonstrated their pivotal role in enhancing the specificity of THz sensing detection methodologies. These include innovative applications such as the precise recognition of dopamine protamine [169], ultrasensitive detection of breast cancer cells facilitated by THz chemical microscopy [170], and the efficient identification of infectious envelope proteins leveraging antibody-based circular sensors [171]. In conclusion, the combination of THz technology and sustainable materials pave the way for advancements in sensing technology, with promising applications in the fields of humidity sensing, environmental hazard factors monitoring, and macromolecule detection.

## THz Biomedical Application

Sustainable materials possess distinctive characteristics such as abundant availability, biodegradability, and excellent mechanical properties. However, certain limitations are accompanied during the fabrication process of the sensing devices using sustainable materials. Sustainable constituents should be conjugated with conductive materials to prepare functional composite materials for the protection of conductive components and are further required to be linked with encapsulation layers to avoid direct contact when serving as substrates or packaging components in clinical applications. Therefore, these materials should be processed into various regenerated forms depending on the research methodology. By leveraging the superior spatial resolution as well as strong penetration ability in biological tissues, detailed THz imaging of tissue structures can be achieved [172]. And the integration of highly biocompatible sustainable materials in THz metamaterials design allows for precise modulation of optical properties, such as amplitude, phase, and polarization [173]. Therefore, these innovations might pave the way for advanced biomedical applications. Herein, the diverse roles of naturally derived sustainable materials enabled THz devices for biomedical applications need to be explored.

### Biomolecule Detection

In recent years, THz biological effects research predominantly relies on the utilization of traditional materials, which resist external influences but are limited by poor degradability. Conversely, sustainable materials, known for their biodegradability, have garnered widespread adoption in the biological field. Researchers are now pivoting toward developing THz sensors with sustainable materials, fostering an innovative and promising synthesis. Currently, the abundant and low-cost sustainable materials fabricated with THz sensors have been applied in biological fields and summarized in Table 4.

**Table 4:** Summary of THz technologies based on sustainable materials in biological fields

Sustainable materials	Functional materials/methods	Fields/targets	Results	References
Aptamer	Hydrogel	α-Thrombin	0.4 pmol L^−1^	[174]
CNFs	Biomimetic aerogels	–	–	[158]
Gelatin	Gelatin embedding	Tissues	–	[175]
Antigen–antibody	Label-free detection	Micro-imaging	–	[176]
Antibody	Antibody and AuNPs	CRP&SAA	1.0 pmol L^−1^	[177]
Antibody	Toroidal resonant modes	Glucose	24.2 pg mL^−1^	[171]
Microcystin aptamer	Microfluidic chip	Microorganism	–	[178]
Aptamer-HB5	Meta-surface-aptamer	Bio-medicine	0.1 ng mL^−1^	[35]
Antibody-CEA	AuNPs-antibody	Bio-medicine	0.1 ng mL^−1^	[179]
Antibody	LQA mode	Biomarkers	3.0 pg mL^−1^	[180]
Aptamer	TCM	Cancer cell	–	[170]
Antibody	GNP and EGFR antibodies	EGFR	10.0 pmol L^−1^	[181]
Aptamer	Graphene-CASR	Detection of microorganism	100.0 nmol L^−1^	[165]
Aptamer	Aptamer and Fe_3_O_4_@Au nanocomposites	Staphylococcus aureus	4.78 × 10^2^ CFU mL^−1^	[182]
SF	Compound material-based SF	Implantable device	–	[183]
SF	MMs-based SF	Living tissues	–	[184]
MCC	Film coatings	Pharmaceutical tablets	–	[185, 186]
Cellulose	Carbon/cellulose composite layer	Wearable electronics	–	[136]
Silk	Silk foam	Biomedical and agro-alimentary industries	–	[187]

*CASR* complementary asymmetry split ring, *CEA* carcinoembryonic antigen, *AuNPs* Au nanoparticles, *LQA* lattice modulated quasi-anapole, *CNFs* cellulose nanofibers, *SK* silk fibroin, *MCC* microcrystalline cellulose, *TCM* THz chemical microscope, *EGFR* epidermal growth factor receptor, *CRP&SAA* c-reactive protein and serum amyloid A

**Fig. 7:**
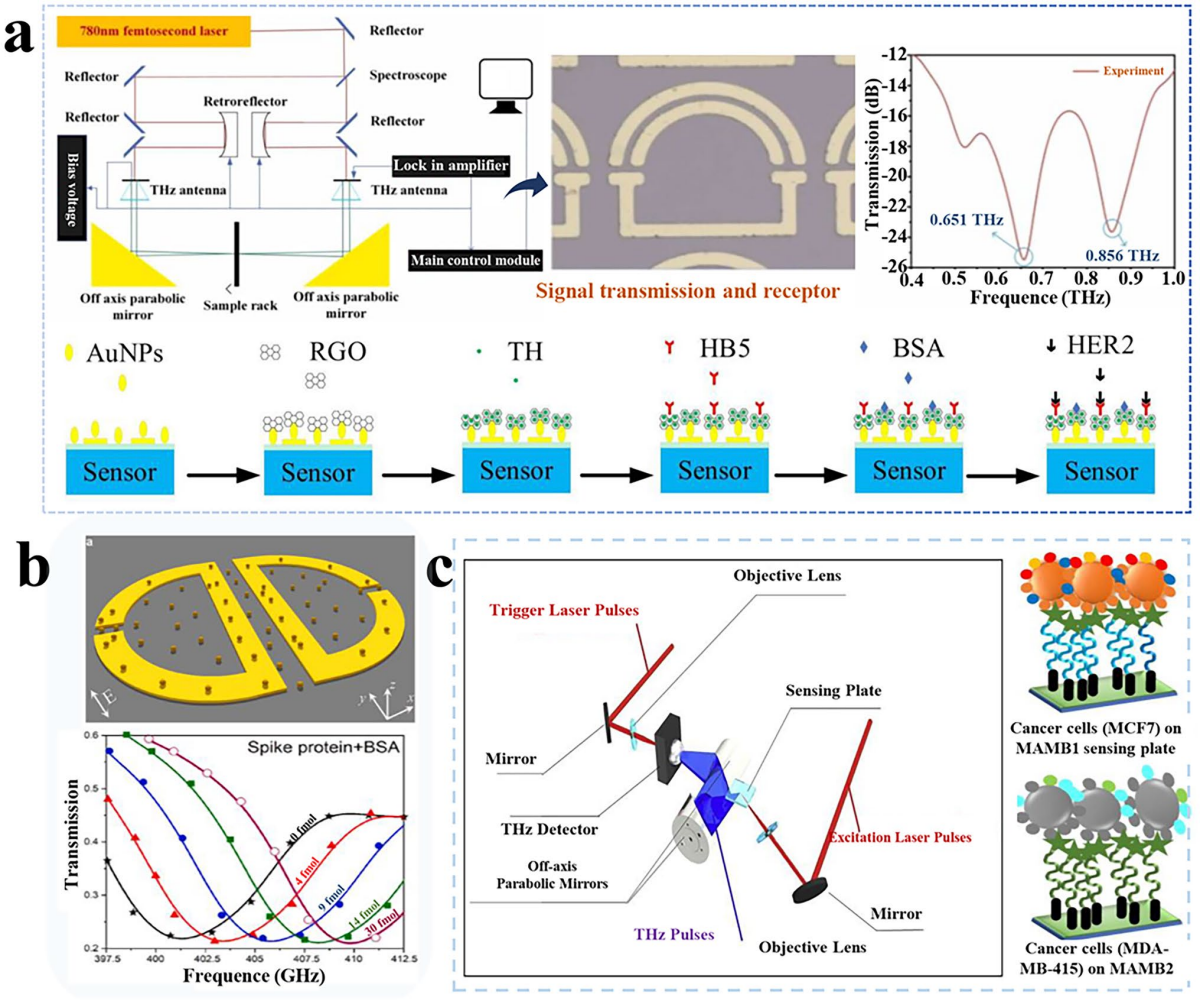
Application and detection of biomolecule, cell, and micro-organism-based THz biosensor with sustainable materials. **a** THz biosensor signal transmission, reception process, and results. Reproduced with permission [35]. Copyright 2022, Elsevier. **b** A microfluidic platform with THz meta-surface chips and Aβ1-42 antibody. Reproduced with permission [191]. Copyright 2021, Elsevier. **c** A sensing plate combined with mammaglobin B1 and corresponding mammaglobin A2. Reproduced with permission [170]. Copyright 2019, Elsevier

In this context, a method was proposed by Zhou et al. [174] for THz metamaterial biosensor based on the functionalization of aptamer hydrogel. This biosensor leverages in-situ polymerization of a specific ligand onto a silanized metamaterial substrate, yielding an aptamer hydrogel with a porous network architecture. The biosensor enabled trace detection of human α-thrombin in a highly absorbent water medium, this biosensor exhibited remarkable sensing capabilities and achieved a LOD as low as 0.4 pM. Besides, the application of aptamer-HB5 was developed for constructing the THz meta-surface biosensor, which introduced a 500 nm thick silicon dioxide spacer between metal split ring resonators and previous silicon substrates as depicted in Fig. 7a. The designed THz biosensor features dual resonant frequencies, with high-frequency resonance and provides superior selectivity [35].

However, silanized materials resist biodegradation and affect the environment. To address this, a sustainable material-based CNF as a film was proposed due to its biodegradability composition of cellulose fibers, hemicellulose, and lignin [117]. THz technology served as a medium for the collection of characteristic signals. For example, a self-supporting thin film was formed by integrating BC with the conductive poly(3,4-ethylenedioxythiophene)/poly(styrenesulfonate) polymer and enables responsiveness to THz regime. The data indicate a significant augmentation in the imaginary dielectric constant of BC, accompanied by a conductivity boost from 0.50 to 1.26 S cm^−1^ within the 0.3–2.8 THz spectrum [188]. This high-dielectric BC exhibits reduced biological tissue rejection, and enhanced biomolecule capture and recognition capabilities, showing an improved sensitivity [189]. Fan et al. [175] introduced an innovative THz imaging and spectral analysis technique that significantly extends tissue preservation time by up to 35 h via gelatin embedding. The absorption coefficient and refractive index were calculated with 6% and 5% at 0.3 THz, which demonstrated the stability of embedded gelatin during the measurement process. This method minimizes alterations in optical parameters, mitigating the impact of sample characteristics.

### Cell and Microorganism Detection

To enhance the selectivity of THz functional devices in cell and virus detection, sensing elements that can specifically recognize biomolecules have also been widely studied [190]. Currently, sustainable bio-materials with excellent biodegradability are used as recognized elements for THz sensors [158] such as specific recognition elements aptamer and antibodies being the most prominent and widely used materials owing to their high specificity, controllable water solubility, and stability. Ahmadivand and coworkers [191] proposed a miniaturized plasmonic immunosensor using monoclonal antibodies on the surface of gold nanoparticles using quasi-infinite meta-surface technology. The antibody was specific to spike protein SARS-CoV-2 virus detection (Fig. 7b). The calculated LOD was extremely low (4.2 fM). Besides, the related reports have verified that the biomolecules possessed extensive applications in the THz sensing field [192, 193]. For example, a microfluidic platform was constructed by decorating Aβ1-42 antibody using a covalent binding method on THz meta-surface chips, which could improve the sensitivity by approximately 100 times compared to other non-specific proteins [194]. Antibody-assisted THz metamaterial biosensor was proposed to detect concentrations of carcinoembryonic antigen, and the result showed that its resonant frequency shift was more significant than those without decorations [179]. Similarly, a sensing plate has been designed, which combines with mammaglobin B_1_ and corresponding mammaglobin A_2_ to effectively monitor the amplitude changes of THz signals as shown in Fig. 7c. The LOD of this sensor is estimated as low as 100 cancer cells in a sample of 1 μL, demonstrating significant progress for timely and ultrasensitive breast cancer cell detection [170]. Furthermore, the integration of sustainable materials like aptamers and antibodies has led to notable improvements in microbial and toxins detection accuracy, exemplified by the high-sensitivity detection of microcystin LR cyanotoxin [178], rapid aptamer nanocomposite-based detection of Staphylococcus aureus [182], and the development of Au nanofilm-based immunosensors for chloramphenicol in milk [195].

### Clinical Medical Treatment

The applications of biomolecules occupy a significant position in THz sensing fields. On the one hand, THz spectrometric approaches are exceptionally suitable for biomedical sensing due to their non-destructive nature and molecular fingerprinting capability. On the other hand, bio-materials can not only enhance the sensitivity and specificity of THz sensing devices but also provide high medical contrast imaging results by leveraging the tunability of biomolecules [196]. Besides, sustainable materials can exert distinctive superiority in special fields including medicine and biology due to their widespread availability and biodegradability [197]. Therefore, rapid and sensitive detection of various biomolecules and exploration of biomolecule applications based on sustainable materials, are crucial for clinical trials. For biomolecule applications, materials should be designed with the following characteristics: (1) maintaining bioactivity and ensuring payload safety; (2) producing non-toxic by-products during processing; (3) being easy to process, biocompatible, and customizable in terms of degradability.

**Fig. 8:**
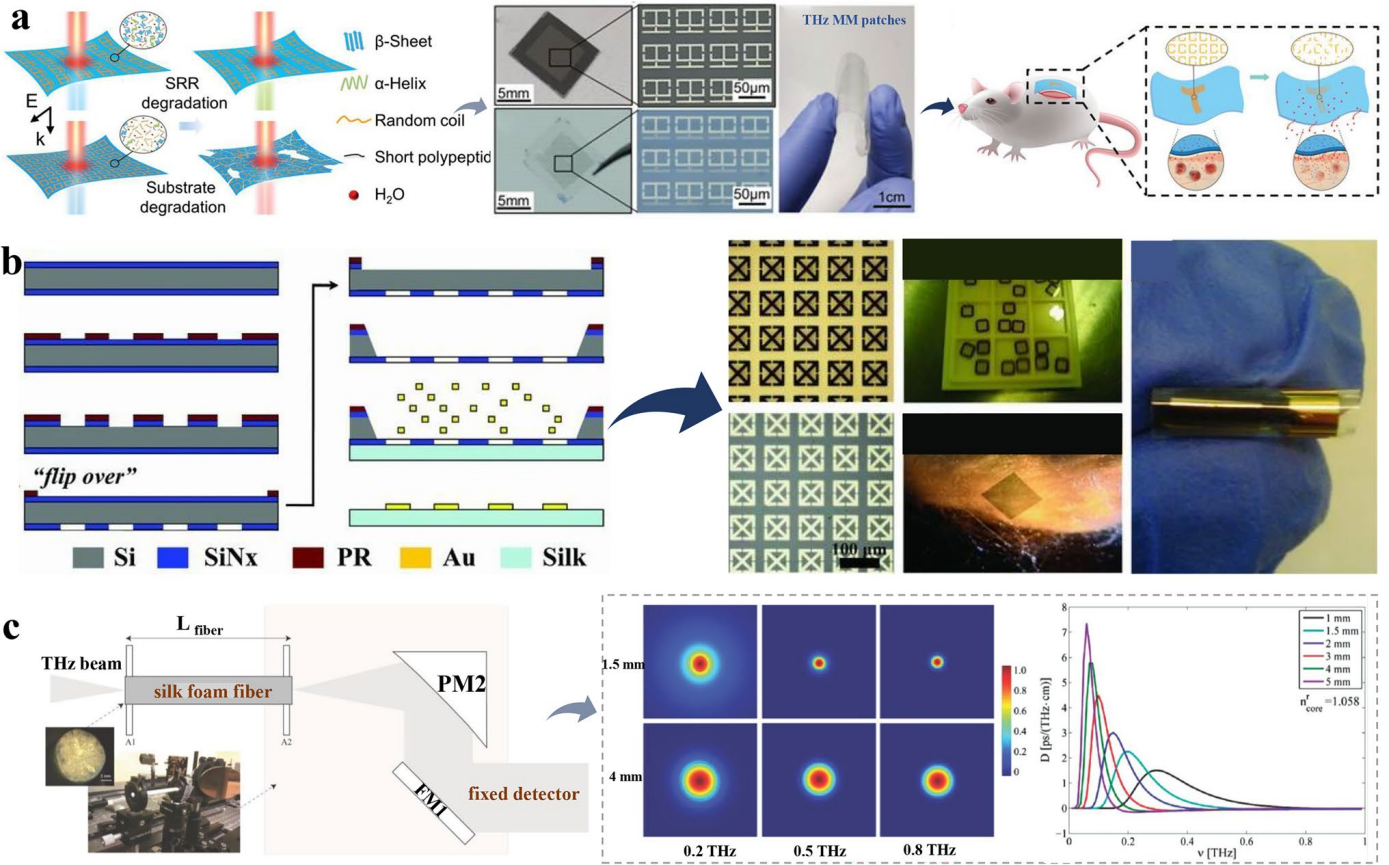
Schematic illustration of the biological effect and medicine treatment based on sustainable materials. **a** Implantable and absorbable therapeutic THz metamaterial patches. Reproduced with permission [184]. Copyright 2020, Wiley. **b** Innovated of an implantable detector with silk and minimal precious metals. Reproduced with permission [183]. Copyright 2010, Wiley. **c** Designing silk foam with two different densities for the biological fluids collection [187]. Reproduced with permission. Copyright 2014, Wiley

THz biosensors based on sustainable materials have enormous potential applications in medical and clinical cases. Tao’s groups [198] designed a THz thin film sensing material based on a split ring resonator (SRR), and monitored the changes in THz time-domain spectral transmission by doping SF. A filament size of 1.35 g cm^−3^ can induce a resonance displacement of 5 GHz. SF is widely spread in clinical medicine due to its degradability and biocompatibility. Considering its distinctive functionality, Sun et al. developed a silk-based metamaterial device capable of loading antibiotics as degradable antibacterial skin patches. Its controllable water solubility and adjustable degradation rate enable real-time monitoring of drug release. As presented in Fig. 8a, the flexibility of silk-based materials allows THz metamaterial patches to adhere effectively to infected mouse surface wounds. Moreover, these implantable and absorbable therapeutic THz devices eliminate the need for retrieval post-implantation, facilitating in vivo sensing and in-situ treatment [184]. Besides, THz technology shows fast response and can perform real-time dynamic detection, which can be utilized for food safety and drug quality control [199]. For example, the photoelectric properties of THz technology are considered as a promising character, it can be used for the bio-issues detection when conjugating with sustainable materials. The group of Omenetto [183] innovated implantable functional devices, using the unique biocompatibility and embeddability of silk fused with minimal precious metals. This device excels in detecting electromagnetic signatures of contrast agents or facilitating biological tracking (Fig. 8b). Notably, the electromagnetic response of surrounding tissues can be seamlessly tuned to the change in metamaterial resonant frequency by scaling its dimensions, a process validated through experiments on porcine muscle tissue slices. Cellulose, as another sustainable material abundant in nature, has shown great potential in the preparation of THz sensors for biological clinical treatment and monitoring. Dong et al. [185] designed a tablet core composed of pure microcrystalline cellulose (MCC), investigating the permeability of coated tablets and the porosity of medium using THz pulse imaging (TPI) technology. The study indicated that the dissolution rate of uncoated tablets decreases by 99.46% before the coating layer is fully dissolved. This finding suggested that the porosity of core influences the coating quality, which affects its dissolution performance. To further study MCC influence on drug release, Zeitler’s group [186] prepared flat pure MCC tablets with a thickness ranging from 1.5 to 1.6 mm, and a thin film coating layer of 80–120 μm was applied on the surface. The utilization of TPI research demonstrated that the gelation of polymers can limit the transportation in capillaries. Moreover, these materials exhibit significant potential in wearable energy storage devices, promoting the detection of physical and biological signals. The flexible sensing device, designed with this 3D composite layer, experiences slight extension or compression, leading to notable changes in carrier density, to be used for remote monitoring of patient conditions [136]. The necessity to explore materials that are perfectly suitable for human skin/body and prevent breakage suggests the development of silk foam with capillary effect, like materials proposed by Guerboukha team [187]. In their work, silk foam with two different densities of fiber core and cladding area was utilized to collect various biological fluids, which was much higher than standard plastics in biosensors because of the porous structure of silk foam (Fig. 8c). These sustainable materials can be effectively applied in the biological and medical field, which offer basic support for the necessary properties of THz sensing devices. Different outputs can be achieved by combining these sustainable materials in various ways. Furthermore, the designed devices are degradable and resorbable in vivo and provide a highly promising power source for biomedical applications.

## Conclusions and Future Perspective

In conclusion, this review highlights the properties and structures of sustainable materials including plant-based materials, protein-based materials, hydrocarbon polymer materials, etc. It also summarizes the development of THz sensing protocols based on sustainable materials. Sustainable materials are primarily employed in the development of biosensing platforms, medical therapies, wireless communication, and information transmission, while also encompassing the creation of platforms for humidity sensing, as well as hazard substances detection. These materials offer significant advantages, such as abundant availability, biocompatibility, programmable biodegradability, and environmental affinity of sustainable materials. This review also covers the applications of THz functional devices based on sustainable materials using biorecognition elements for harmful substance detection, and environmental monitoring, among other areas in various forms such as films, composite materials, hydrogels, and aerogels. Despite significant progress in THz functional devices based on sustainable materials, including low-loss THz transmission devices, high performance THz modulators, high sensitivity sensors [200], several challenges persist that need to be addressed. One key issue is the limited tunability of sustainable materials in the THz band. To overcome this requires the design of materials exhibiting adjustable conductivity and nonlinear optical responses. For instance, doping conductive materials into cellulose or chitosan can enhance the tunability of devices [201]. Secondly, the structure design and micro-nano processing of sustainable materials are required to be promoted. Artificial intelligence (AI) technology can be applied in speedy and precise design of sustainable materials-based THz functional devices [202, 203]. Furthermore, inaccurate preparation of these sustainable materials might lead to erroneous results or imprecise drug delivery sites in practical THz functional device applications. To resolve this issues, deep learning techniques [204], such as convolutional neural networks (CNN), can be employed to construct a CNN-gated recurrent unit (GRU) model [205] for calibration experiments, ensuring results that align with expectations.

From a materials perspective, sustainable materials exhibit characteristics such as resource availability and environmental friendliness. For instance, nitrogen-doped carbon nanotubes and bio-based polymers possess high electrical conductivity and broad-band optical absorption properties, making them outstanding performers in the THz frequency range. However, the complexity of manufacturing these materials limits their wide commercial applications. Furthermore, changes in environmental conditions can easily make THz functional devices unstable. Additionally, when sustainable materials are used as substrates for THz functional devices, essential functional composites such as metals are still required. Thus, there is significant room for improvement in sustainable materials through methods such as chemical modification and interface structure design to endow them with unique properties.

From the perspective of functional device construction, THz functional devices integrated with sustainable materials exhibit high sensitivity and rapidity. These functional devices are designed to be more compact and wearable. However, the design requires precise process control, which increases the complexity of the manufacturing process. Another challenge is that sustainable materials may experience degradation under extreme conditions, such as high humidity or high temperatures, affecting their efficacy in practical settings [206]. This issue is particularly critical in clinical medical applications, where THz functional devices based on sustainable materials might require secondary surgeries due to the inability to provide continuous energy. Future research should focus on developing sustainable THz functional devices that integrate energy storage and sensing capabilities.

Regarding the application perspective, THz functional devices fabricated with sustainable materials exhibit significant potential in diverse fields, including security, medical diagnostics, environmental monitoring, and satellite communication. These functional devices provide high-performance detection capabilities, addressing various application requirements while minimizing dependence on harmful substances, thus reducing environmental pollution and resource consumption. However, their implementation in practical applications suffers from several challenges. For example, in the medical field, the market acceptance of new technologies and materials requires time. Additionally, in environmental monitoring, certain functional devices pose recycling challenges, and THz functional devices integrated with functional composites may contribute to environmental pollution. Therefore, the development of THz functional devices utilizing sustainable materials necessitates ongoing exploration and research into fully degradable, recyclable, and environmentally safe sensing devices. This comprehensive review offers a pioneering avenue for the development of THz functional devices, exploiting the unique properties of sustainable materials. By harnessing the inherent benefits of sustainable materials and tapping into the vast potential of THz functional devices across various domains, the methodologies discussed in the current work hold promise for advanced medical diagnostics, biological research, and communication technologies.
